# The selective degradation of sirtuins via macroautophagy in the MPP^+^ model of Parkinson’s disease is promoted by conserved oxidation sites

**DOI:** 10.1038/s41420-021-00683-x

**Published:** 2021-10-12

**Authors:** Marius W. Baeken, Mario Schwarz, Andreas Kern, Bernd Moosmann, Parvana Hajieva, Christian Behl

**Affiliations:** 1 Institute for Pathobiochemistry, The Autophagy Lab, University Medical Center of the Johannes Gutenberg University, Mainz, Germany; 2 Institute for Pathobiochemistry, Evolutionary Biochemistry and Redox Medicine, University Medical Center of the Johannes Gutenberg University, Mainz, Germany; 3 Institute for Pathobiochemistry, Cellular Adaptation Group, University Medical Center of the Johannes Gutenberg University, Mainz, Germany; 4 Present Address: Nucleic Acid Chemistry and Engineering Unit, Okinawa Institute of Science and Technology Graduate University, Onna, Okinawa, 904 0495 Japan; 5 Present Address: Institute for Molecular Medicine, MSH Medical School, Hamburg, Germany

**Keywords:** Proteolysis, Neurodegeneration

## Abstract

The sirtuin (SIRT) protein family has been of major research interest over the last decades because of their involvement in aging, cancer, and cell death. SIRTs have been implicated in gene and metabolic regulation through their capacity to remove acyl groups from lysine residues in proteins in an NAD^+^-dependent manner, which may alter individual protein properties as well as the histone–DNA interaction. Since SIRTs regulate a wide range of different signaling cascades, a fine-tuned homeostasis of these proteins is imperative to guarantee the function and survival of the cell. So far, however, how exactly this homeostasis is established has remained unknown. Here, we provide evidence that neuronal SIRT degradation in Parkinson’s disease (PD) models is executed by autophagy rather than the proteasome. In neuronal Lund human mesencephalic (LUHMES) cells, all seven SIRTs were substrates for autophagy and showed an accelerated autophagy-dependent degradation upon 1-methyl-4-phenylpyridinium (MPP^+^) mediated oxidative insults in vitro, whereas the proteasome did not contribute to the removal of oxidized SIRTs. Through blockade of endogenous H_2_O_2_ generation and supplementation with the selective radical scavenger phenothiazine (PHT), we could identify H_2_O_2_-derived species as the responsible SIRT-oxidizing agents. Analysis of all human SIRTs suggested a conserved regulatory motif based on cysteine oxidation, which may have triggered their degradation via autophagy. High amounts of H_2_O_2_, however, rapidly carbonylated selectively SIRT2, SIRT6, and SIRT7, which were found to accumulate carbonylation-prone amino acids. Our data may help in finding new strategies to maintain and modify SIRT bioavailability in neurodegenerative disorders.

## Introduction

The sirtuin (SIRT) protein family and their influence on epigenetics and metabolism have been studied extensively. As such, several SIRTs were correlated to longevity, even across species, and their decline is considered a hallmark of senescence [1]. Indeed, SIRT-activating small molecules are actively pursued in human anti-aging trials [2]. In cancer, however, SIRTs show an astonishing duality, as they can act as either tumor suppressors or oncogenes [3].

SIRTs may be considered peculiar as they require NAD^+^ as a cofactor but do not reduce it the way other proteins usually do. Instead, SIRTs cleave the nicotinamide group of the ADP-ribose unit and use the latter as an acceptor for the acyl-group they remove from lysine residues [4]. This, however, puts SIRTs in immediate competition with other enzymes that require NAD^+^. If, for example, poly ADP-ribose polymerases consume high levels of NAD^+^, mitophagy is compromised through SIRT inhibition [5, 6].

Thus, proper homeostasis of SIRTs is essential to maintain cellular prosperity. Homeostasis of SIRTs, like the homeostasis of the entire proteome, is maintained by their assembly and disassembly, folding, refolding, and degradation [7]. The mechanisms by which dysfunctional SIRTs or excess SIRTs are degraded, however, are only marginally characterized [8].

Aging cells frequently accumulate oxidized biomolecules, which is especially critical in neurons representing a postmitotic, none-replenishable cell type. Indeed, the accumulation of oxidative damage is a hallmark of different neurodegenerative disorders like Parkinson’s disease (PD), Alzheimer’s disease, or amyotrophic lateral sclerosis [9–11]. Autophagy has been implied as a major contributor to remove oxidized and cross-linked proteins [12]. Indeed, previous studies revealed that aging cells might preferentially adopt autophagy to maintain protein homeostasis [13].

SIRTs indeed do contribute to autophagy by directly regulating different components of the autophagic machinery through deacylation. For example, SIRT1 reportedly regulates cytosolic availability of microtubule-associated protein 1 light chain 3 beta (MAP1LC3B, also known as LC3 or LC3B), an autophagosomal component of major importance [14, 15]. Similar observations were published in vivo concerning the acetylation status of ATG5 and ATG7 [16].

In this study, we aimed to investigate systematically how and to what extent increased endogenous reactive oxygen species (ROS) affect human SIRTs, and by which means oxidized or damaged SIRTs are removed or salvaged.

## Results

### Protein levels of most human SIRTs decrease under oxidative stress

First, we investigated how SIRT protein levels would respond to increased endogenous ROS, induced by the PD models 1-methyl-4-phenylpyridinium (MPP^+^), rotenone, and paraquat in vitro, using LUHMES cells. MPP^+^ and rotenone share a similar pathomechanism, as both inhibit the complex I of the mitochondrial respiratory chain. Paraquat mostly acts as a cytosolic redox-cycler [17]. To verify whether SIRT dynamics were related to increased ROS, we included three control treatments. First, the uncoupler FCCP induces mitochondrial stress without immediately increasing ROS formation. Second, we applied the antioxidant phenothiazine (PHT), an established, selective radical scavenger in PD models [18–20]. Finally, a PHT control group was monitored.

**Fig. 1:**
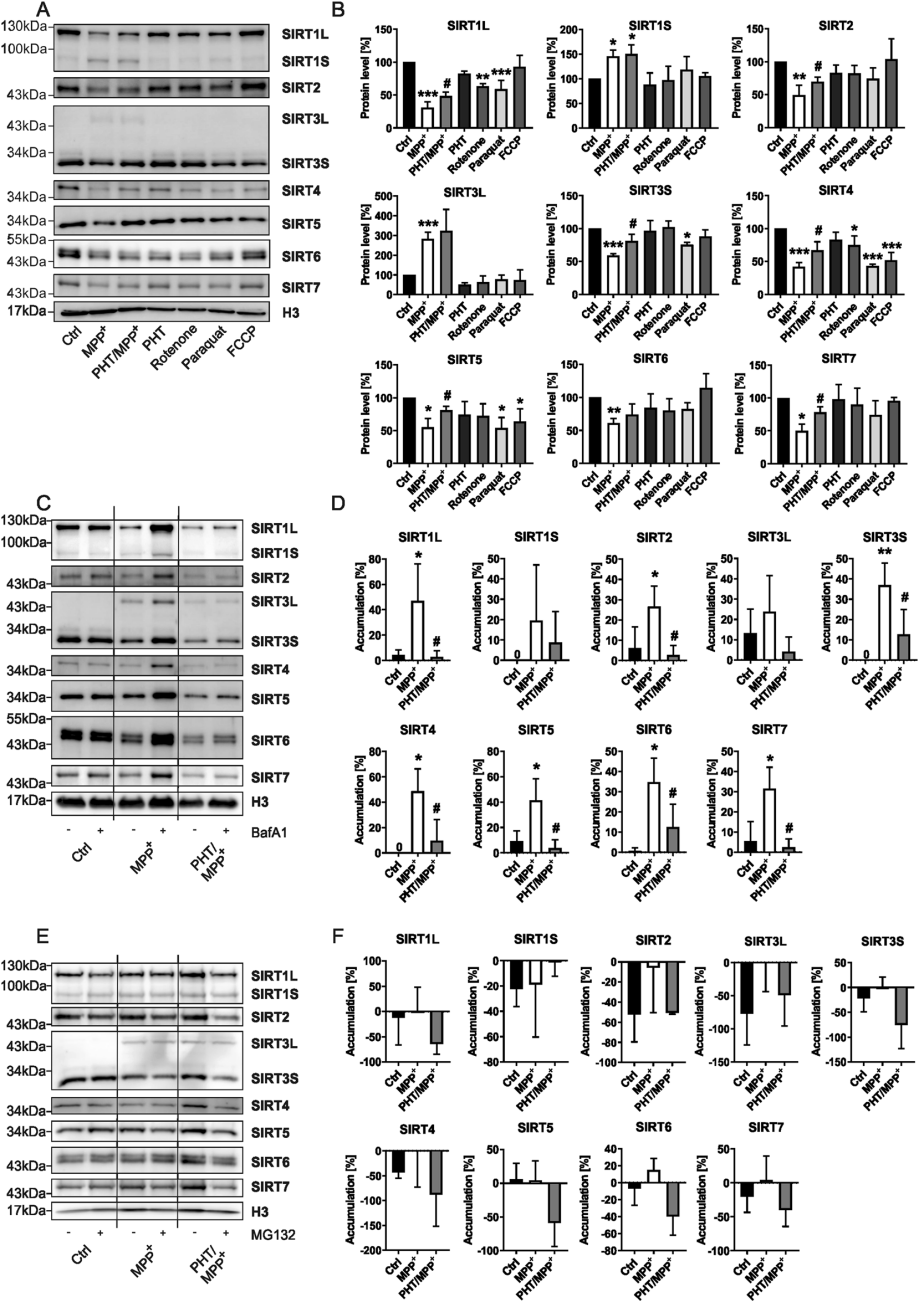
Induction of endogenous ROS provokes a disruption of SIRT protein homeostasis promoted by their autophagic degradation. **A** Representative immunoblots of all SIRTs were obtained from differentiated LUHMES cells after 48 h treatments with MPP^+^ (10 µM), PHT (20 nM), rotenone (10 nM), paraquat (100 µM), or FCCP (1 mM). H3 was used as a loading control. **B** Bar graphs showing the total SIRT protein levels of the blots presented in a. “*” indicates significant differences compared to the control group, while “#” indicates significant differences compared to the MPP^+^-treated group. Symbol number indicates the grade of significance with *p < 0.05, ** *p* < 0.01, and ***p < 0.001. **C** Representative immunoblots of all SIRTs obtained from differentiated LUHMES cells after 48 h treatments with MPP^+^ (10 µM) and PHT (20 nM) with and without BafA1 (500 nM) supplementation for 4 h. H3 was used as a loading control. **D** Bar graphs showing the SIRT accumulation after BafA1 mediated block of autophagic degradation seen in the blots presented in **C**. “*” indicates significant differences compared to the control group, while “#” indicates significant differences compared to the MPP^+^-treated group. Symbol number indicates the grade of significance with *p < 0.05 and **p < 0.01. **E** Representative immunoblots of all SIRTs were obtained from differentiated LUHMES cells after 48 h treatments with MPP^+^ (10 µM) and PHT (20 nM) with and without MG132 (10 µM) supplementation for 24 h. H3 was used as a loading control. **F** Bar graphs showing the SIRT accumulation after MG132 mediated block of proteasomal degradation seen in the blots presented in **E**.

MPP^+^ at sublethal concentration caused a significant decrease of the main isoforms of all SIRTs (SIRT1–7) in LUHMES cells (Fig. 1A, B). With the exception of SIRT6, these decreases were significantly attenuated by concomitant PHT administration. Two SIRTs presented as long and short isoforms, SIRT1 as SIRT1L at ~130 kDa (main) and SIRT1S at ~75 kDa (minor), and SIRT3 as SIRT3L at ~43 kDa (minor) and SIRT3S at ~28 kDa (main). Interestingly, both minor isoforms, SIRT1S and SIRT3L, were significantly increased in MPP^+^-treated cells and unaffected by PHT co-treatment. Rotenone treatment caused a significant decline in SIRT1L and SIRT4, while paraquat additionally affected SIRT3S and SIRT5 levels significantly. Interestingly, the non-oxidative uncoupler FCCP induced a selective loss of SIRT4 and SIRT5, possibly through the induction of mitophagy [21]. Sole PHT treatment never affected SIRT protein levels significantly.

### SIRTs are subject to autophagic degradation upon induction of oxidative stress

To clarify the origin of ROS-mediated SIRT protein loss, we focused on the MPP^+^ and MPP^+^/PHT treatment groups since MPP^+^ generally showed the most pronounced effects on SIRT levels (Fig. 1A, B). To inhibit lysosomal acidification and thus degradation of autophagosomal cargo, we added Bafilomycin A1 (BafA1) for the last 4 h of the experiment. Under basal conditions, SIRTs showed no significant accumulation in response to BafA1. However, under MPP^+^ treatment, all previously downregulated SIRTs significantly accumulated in response to BafA1. Again, the minor isoforms SIRT1S and SIRT3L did not recapitulate this pattern. PHT/MPP^+^-treated cells demonstrated substantially lowered SIRT accumulation throughout (Fig. 1C, D), indicating that ROS were the general cause of the MPP^+^-triggered degradation of SIRTs.

Subsequently, we tested whether SIRTs would accumulate when proteasomal degradation was inhibited by MG132. However, no significant effects could be observed (Fig. 1E, F), suggesting that the proteasome does not play a relevant role in SIRT turnover in LUHMES cells. Effective proteasomal inhibition by MG132 was confirmed by measuring the general accumulation of poly-ubiquitinated proteins (Supplementary Fig. 1a, b).

### Autophagic SIRT degradation requires oxidative stress

**Fig. 2:**
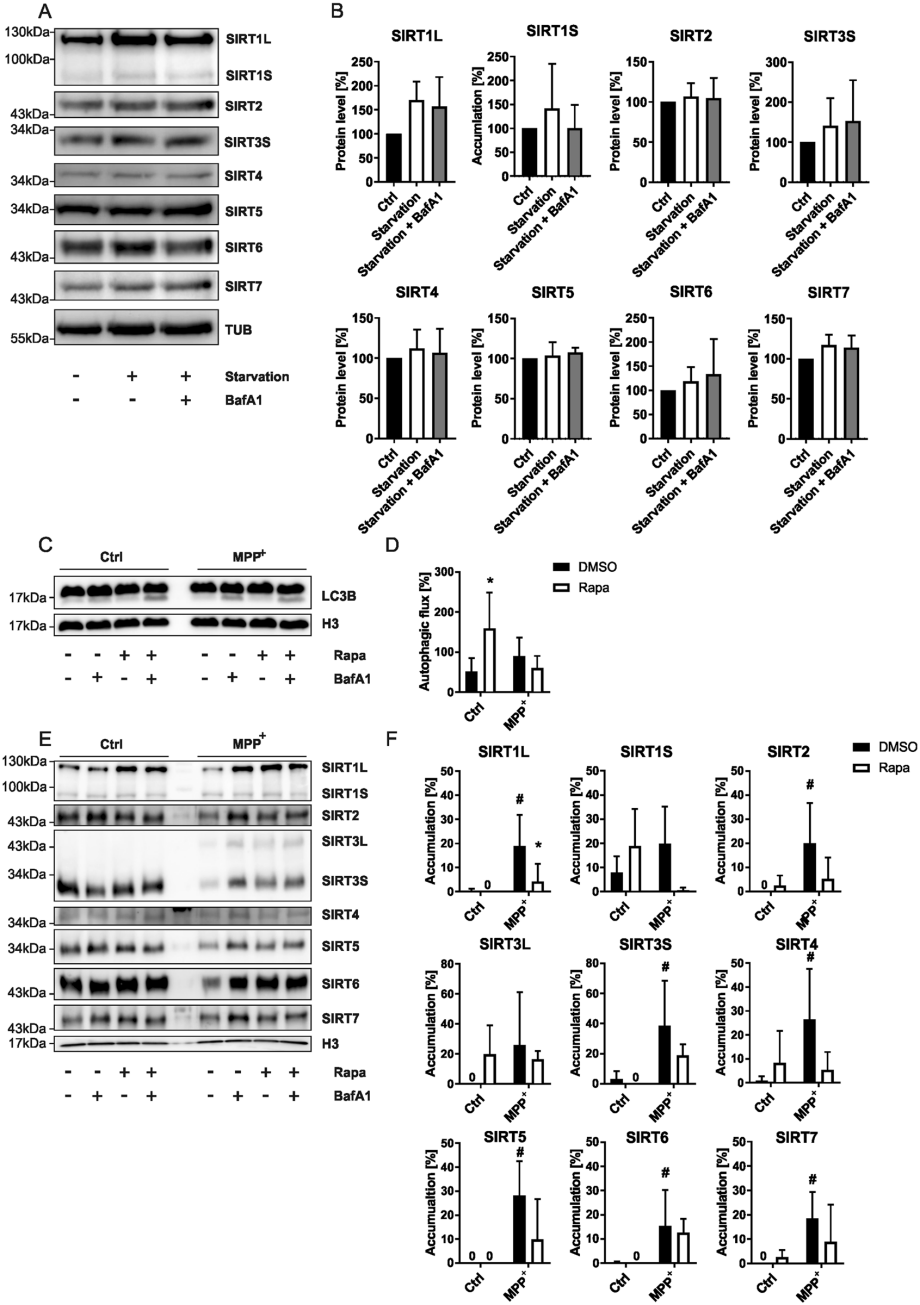
SIRT degradation is dependent on an increase in oxidative stress. **A** Representative immunoblots of all SIRTs obtained from differentiated LUHMES cells after 4 h of starvation in EBSS with and without BafA1 (500 nM) supplementation for 4 h. TUB was used as a loading control. **B** Bar graphs showing the total SIRT protein levels of the blots presented in **A**. **C** Representative immunoblots of LC3B obtained from differentiated LUHMES cells after 48 h treatments with MPP^+^ (10 µM) with and without BafA1 (500 nM) and/or rapamycin (500 nM) supplementation for 4 h. H3 was used as a loading control. **D** Bar graphs showing the autophagic flux derived from the blots presented in **C**. “*” indicates significant differences compared to the DMSO control group, symbol number indicates the grade of significance with *p < 0.05. **E** Representative immunoblots of all SIRTs were obtained from differentiated LUHMES cells after 48 h treatments with MPP^+^ (10 µM) with and without BafA1 (500 nM) and/or rapamycin (500 nM) supplementation for 4 h. H3 was used as a loading control. **F** Bar graphs showing the SIRT accumulation after BafA1 mediated block of autophagic degradation seen in the blots presented in **E**. “*” indicates significant differences compared to the control group, while “#” indicates significant differences compared to the DMSO control-treated group. Symbol number indicates the grade of significance with *p < 0.05.

Next, we investigated whether SIRTs become substrates for autophagic-lysosomal degradation under general autophagy-inducing conditions. We subjected LUHMES cells to starvation with and without BafA1. However, we could not observe significant changes regarding any SIRT (Fig. 2A, B).

Consequently, we wanted to examine whether SIRT degradation could be further stimulated when the cells were subjected to increased ROS. However, starvation in combination with MPP^+^ treatment caused a critical decline in LUHMES cell viability. Thus, we chose to induce autophagy pharmacologically by rapamycin (Fig. 2C–F). As expected, rapamycin increased autophagic activity as quantified [22] per the ATG8 paralogue LC3B (Fig. 2C, D) in our control samples. Interestingly, the induction was lost in cells co-treated with MPP^+^. Rapamycin may have been unable to increase the autophagic flux in MPP^+^-treated cells, since MPP^+^ already inhibits mTOR [23]. Thus, the pathway could already be pushed to its limit in this treatment paradigm. Indeed, results in SH-SY5Y cells confirmed that the autophagic flux could no longer be induced after exposure to mild levels (10 µM) of MPP^+^ [24].

Rapamycin had no immediate effects on autophagic SIRT degradation at baseline (Fig. 2E, F). On the other hand, the manifest induction of SIRT degradation upon MPP^+^ treatment was prevented by rapamycin treatment (Fig. 2C, D). However, antagonistic effects of rapamycin towards MPP^+^ mediated toxicity are known [25].

### SIRTs co-localize with autophagosomal structures

**Fig. 3:**
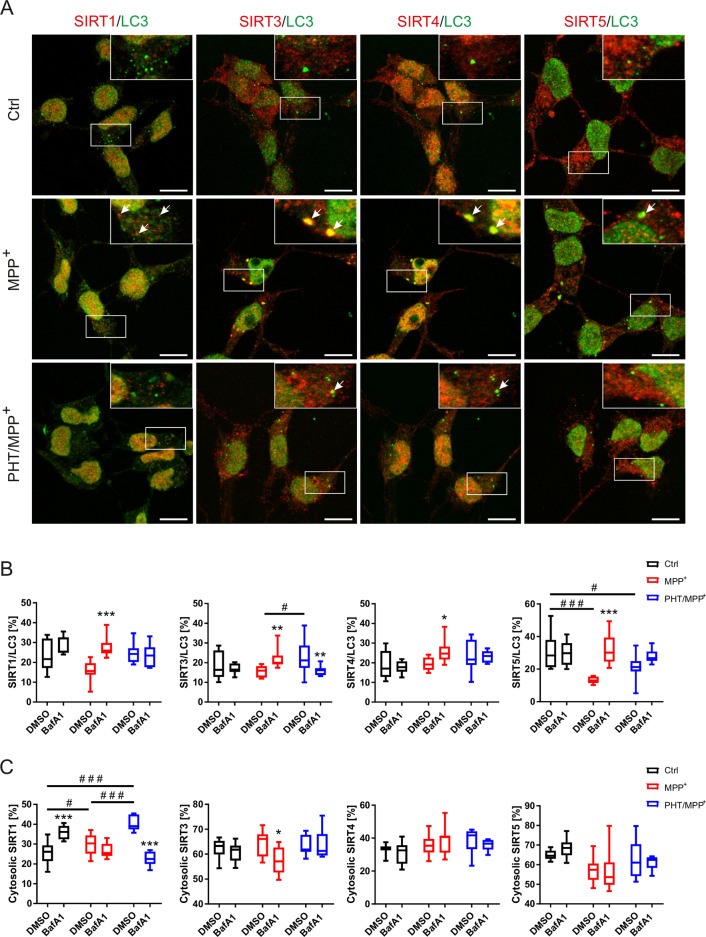
SIRTs accumulate in autophagosomal structures. **A** Representative LSM images of differentiated LUHMES cells treated after 48 h treatment with MPP^+^ (10 µM) and/or PHT (20 nM) with BafA1 (500 nM) supplementation for 4 h with 63x magnification. The upper right corner per image highlights the area indicated by the white rectangle. Stained SIRTs are shown in red and LC3B in green. Scale bars represent 10 µm. Arrows indicate SIRT/LC3B co-localized vesicles. **B** Boxplots describing the cytosolic co-localization of the indicated SIRT with LC3B of images shown in **A**. “*” indicates significant differences caused by BafA1 treatment, while “#” indicates significant differences between the DMSO treated samples. Symbol number indicates the grade of significance with *p < 0.05, **p < 0.01, and ***p < 0.001. **C** Boxplots describing the cytosolic localization of the indicated SIRT of images shown in **A**. “*” indicates significant differences caused by BafA1 treatment, while “#” indicates significant differences between the DMSO treated samples. Symbol number indicates the grade of significance with *p < 0.05 and ***p < 0.001.

To verify whether SIRTs actually co-localize with autophagosomal structures, we performed immunocytochemistry for SIRT1, SIRT3, SIRT4, SIRT5, and LC3B. Analysis was restricted to cytosolic co-localization to exclude nuclear co-localization not related to autophagosomes [14]. Co-localization between cytosolic SIRTs and LC3B was significantly induced in MPP^+^- and BafA1-treated cells (versus MPP^+^-alone treated cells), suggesting higher incorporation of SIRTs into autophagosomal structures (Fig. 3A, B and Supplementary Fig. 2). This effect was reverted by co-treatment with PHT, indicating again an oxidation-related phenomenon. Although recent advances in the field demonstrated the occurrence of LC3B positive, vesicular structures in autophagy-deficient cells [26], their relative absence in our samples not treated with BafA1 would rather suggest a formation of autophagosomes than mere p62 aggregation (Fig. 3A, B and Supplementary Fig. 2).

As expected, the difference between MPP^+^-alone treated cells and MPP^+^/BafA1-treated cells was in part evoked by a loss (of cytosolic) co-localization in MPP^+^-alone treated cells; this effect was significant for SIRT5 and SIRT1. Hence, MPP^+^ would have induced the disappearance of SIRTs from the cytosol by the successful execution of autophagy. In order to probe whether this interpretation was correct and unaffected by nuclear shifts, we also analyzed the relative cytosolic fraction of each SIRT (Fig. 3A, C). None of the SIRTs exhibited a smaller cytosolic fraction under MPP^+^ or PHT/MPP^+^ treatment at baseline, and except for the borderline significant SIRT3, BafA1 treatment caused no shift in MPP^+^-treated cells.

SIRT1 was unique in being redistributed into the cytosol by BafA1 alone, and in being redistributed into the nucleus by PHT. Tentatively, we assign this behavior to an unusually rapid turnover of SIRT1, including a rapidly adjusting equilibrium between nucleus and cytosol. This idea is supported by the fact the SIRT1 was the most heavily affected protein by MPP^+^ treatment (Fig. 1A–D). The rather high levels of the canonically mitochondrial SIRT4 in the nucleus are not unprecedented [27]. Similarly, about 30% of LUHMES cell SIRT5, which has been shown to desuccinylate nuclear proteins [28], was detected in the nucleus (Fig. 3C).

### SIRTs are damaged by H_2_O_2_ and its products

**Fig. 4:**
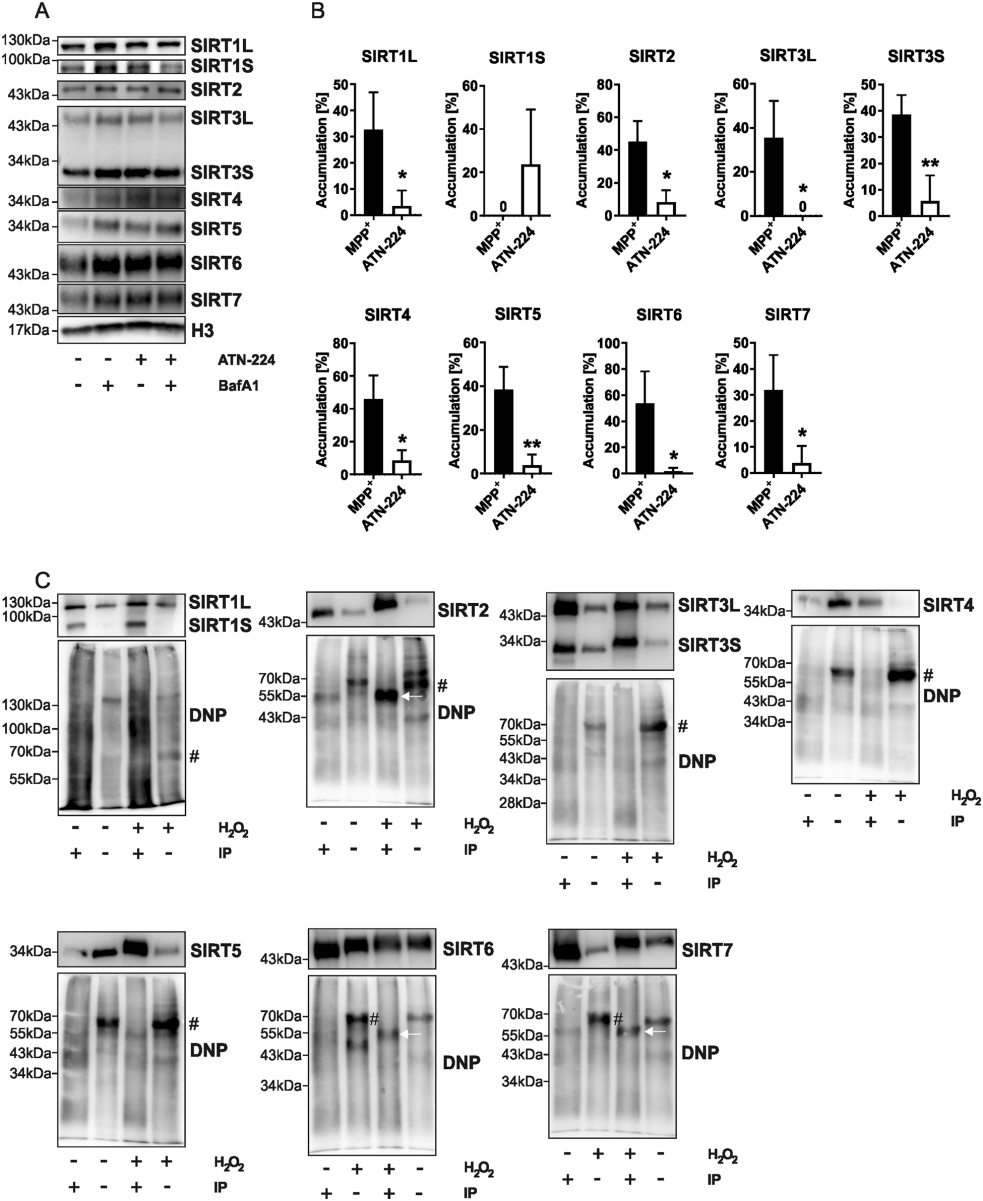
H_2_O_2_, but not superoxide, damages some SIRTs. **A** Representative immunoblots of all SIRTs obtained from differentiated LUHMES cells after 48 h treatment with MPP^+^ (10 µM) with and without BafA1 (500 nM) supplementation for 4 h. ATN-224 (10 µM) was administered over 6 h. H3 was used as a loading control. **B** Bar graphs showing the SIRT accumulation after BafA1 mediated block of autophagic degradation seen in the blots presented in **A**. “*” indicates significant differences compared to the control group, while “#” indicates significant differences compared to the DMSO control-treated group. Symbol number indicates the grade of significance with *p < 0.05. **C** Immunoblots of immunoprecipitated ectopically expressed SIRTs and total protein lysate from HEK-T cells showing carbonylated proteins derivatized with DNP compared to total proteins after 1 h of 20 mM H_2_O_2_ treatment. The upper blot shows the signal generated using a SIRT-specific antibody without prior DNP derivatization, lower blot shows the signal generated using the DNP-specific antibody after DNP derivatization. H_2_O_2_ indicates which samples were submitted to an oxidative environment, IP indicates which lanes were loaded with the IP purified protein post oxidation. A “#” indicates carbonylated proteins at ~70 kDa, while the white arrows point at carbonylated SIRTs.

Which ROS could be the mediators of the observed, increased degradation of SIRTs in response to MPP^+^ treatment? In a first experiment, we administered MPP^+^-treated LUHMES cells with BafA1 and ATN-224, a pleiotropic SOD inhibitor (Fig. 4A, B). ATN-224 alone had no significant effects on SIRT levels or turnover (Supplementary Fig. 3). Interestingly, we obtained similar results for all SIRTs (except SIRT1S), in that ATN-224 caused a reduction of the MPP^+^-mediated degradative accumulation (Fig. 4A, B). This observation seems to rule out any direct oxidizing effect of MPP^+^-induced superoxide on SIRTs. The reported ATN-224-evoked loss of H_2_O_2_ generation may well account for our findings [29].

Endogenous H_2_O_2_ cannot easily be enriched in a similar manner to superoxide in vitro. Thus, we decided to take a different approach and analyzed SIRT protein oxidation by H_2_O_2_ in terms of protein carbonylation of ectopically expressed SIRTs from HEK-T cells (Fig. 4C). SIRT1, SIRT3, SIRT4, and SIRT5 showed no increase in protein carbonylation after high-dose H_2_O_2_ treatment in vitro, whereas SIRT2, SIRT6, and SIRT7 did. Strikingly, three of the four none-carbonylated SIRTs are mitochondrial. This prompted us to look at possible footprints of natural selection towards or away from oxidative resistance in the amino acid composition of SIRTs across the *Bilateria* clade.

### Natural selection favored none-mitochondrial SIRTs that can be regulated through oxidation

Briefly revisiting the phylogeny of the SIRTs (Supplementary Fig. 4) confirmed the described classification of human SIRTs: class I (SIRT1, SIRT2, and SIRT3), class II (SIRT4), class III (SIRT5), and class IV (SIRT6 and SIRT7) [30]. Eukaryotic SIRT5 appeared distant from other eukaryotic SIRTs, but in proximity to those of the archaea. Contrarily, SIRT4 appeared close to SIRTs from Wolbachia, which are related to proto-mitochondria [31].

**Fig. 5:**
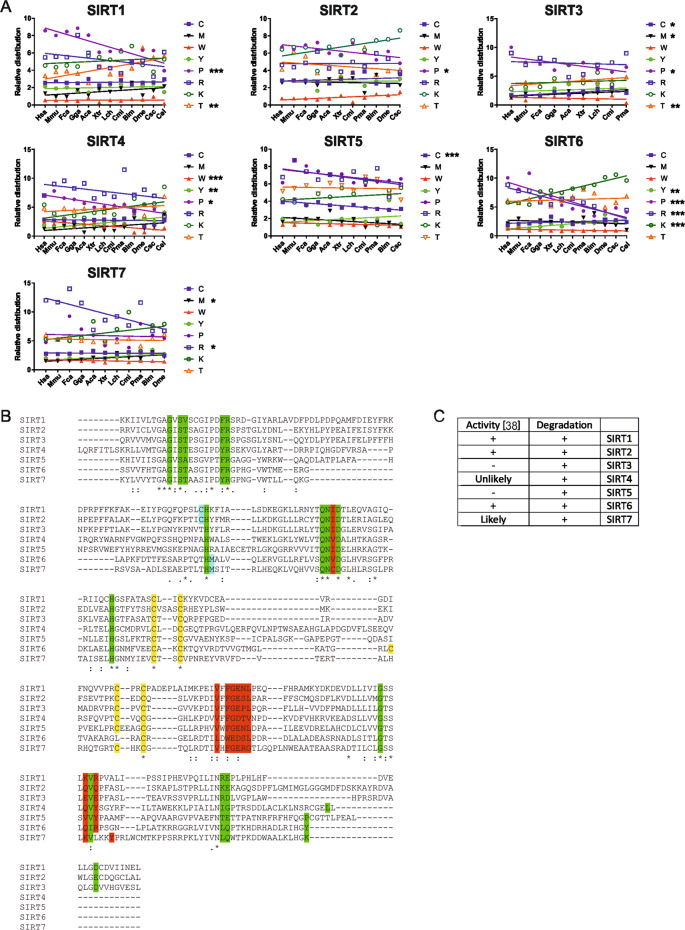
SIRT vulnerability to oxidative stress is conserved. **A** Shift in indicated amino acid compositions of SIRTs in single letter code throughout *bilateria* evolution. Plotted species included *Homo sapiens* (Hsa), *Mus musculus* (Mmu), *Felis catus* (Fca), *Gallus gallus* (Gga), *Anolis carolinensis* (Aca), *Xenopus tropicalis* (Xtr), *Latimeria chalumane* (Lch), *Callorhinchus milii* (Cmi), *Petromyzon marinus*, *Bombus impatiens*, *Drosophila melanogaster*, *Centruroides sculpturatus* (Csc), and *Caenorhabditis elegans* (Cel). For each amino acid, a linear regression was generated and calculated whether the regression showed a significant progression. Symbol number indicates the grade of significance with *p < 0.05, **p < 0.01, and ***p < 0.001. **B** Alignment of the enzymatic centers of human SIRTs. Green color indicates NAD^+^ binding, red substrate binding, yellow the Zn^2+^-tetrathiolate, and blue the possible activity regulating sulfurous amino acids. * indicates identical amino acids, conserved and semi-conserved sites. **C** Table comparing which SIRT’s activity is regulated by oxidation, based on [38], and which SIRT is degraded by autophagy upon oxidation.

Cysteine is arguably the most rapidly oxidized amino acid under standard conditions. Therefore, previous studies have suggested that along with the evolution of aerobic organisms, cysteine (C) residues were selectively depleted [32, 33]. The observed C frequency in human proteins averages ~2.2% [34]. With the exception of SIRT3, which showed a significant decline, human SIRTs generally harbored a (modestly) higher amount of evolutionarily stable C residues (Fig. 5A). Methionine (M) also decreased in SIRT3; a progression only shared by SIRT7. The mitochondrial nature of SIRT3 could have required it to attain a more oxidation-resilient structure. SIRT5, in turn, behaved completely differently, as its generally high C content progressively increased. Next, we looked at the usually antioxidative amino acids tyrosine (Y) and tryptophan (W) [33, 35]. Again, most SIRTs demonstrated no significant changes, except for SIRT4, in which W and Y appeared to be mutually replaced over time (Fig. 5A).

A carbonylation is a terminal form of protein damage that leads to proteasomal degradation [36]. Accordingly, protein carbonyls predominantly arise from relatively stable amino acids such as arginine (R), lysine (K), proline (P), and, to some degree, threonine (T) [37]. Notably, the carbonylated SIRTs all showed net accumulation of these amino acids (higher P in SIRT2; higher P and R, but lower K in SIRT6; higher R in SIRT7) (Fig. 5A).

Hence, the evenly distributed susceptibility of SIRTs to oxidant-induced autophagic degradation most likely arises from the oxidation of their C residues. Indeed, a conserved Zn^2+^-tetrathiolate was reported highly oxidation-prone (Fig. 5B) [38]. Interestingly, an alignment of the catalytic domain of human SIRTs revealed sulfurous amino acids next to an NAD^+^-binding histidine exclusively in none-mitochondrial SIRTs (Fig. 5B). A comparison with recently published data [38] indicates that the reported activity pattern would fit oxidation of these sulfurous amino acids, while the autophagic degradation pattern reported here may originate from plain oxidation of the tetrathiolate (Fig. 5C).

## Discussion

The homeostasis of SIRT expression is of high biomedical interest since essentially all SIRTs appear to antagonize aspects of aging [39–45] or neurodegeneration [46]. These observations are, however, still discordant in invertebrates [47, 48]. Furthermore, similar results were obtained where a general overexpression of SIRT1 did not lead to increased longevity in mice. Yet, overexpression limited to the brain (BRASTO) caused a significantly later onset of senescence [39]. So far, the knowledge about the general regulation of SIRT activity in mammalian cells has remained incomplete. Here, we provide data indicating that endogenous ROS in form of H_2_O_2_ or its decomposition products broadly oxidize SIRTs and induce their autophagic degradation in dopaminergic neuronal cells in models of PD. This is also supported by the absence of degradation through SOD inhibition or its presence upon paraquat administration.

**Fig. 6:**
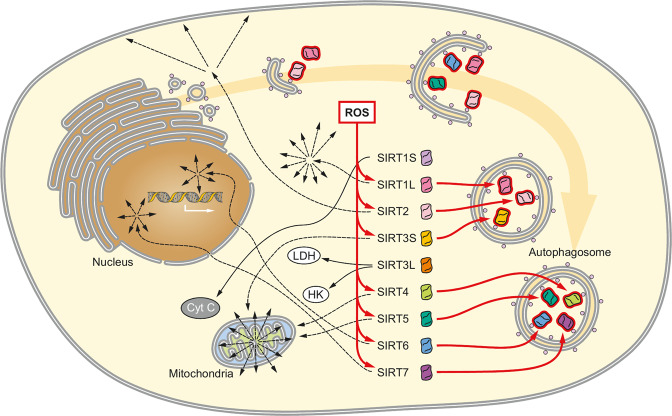
Graphical model of the SIRT status in LUHMES cells subjected to chronically increased oxidative stress. Most SIRTs become substrates for autophagic degradation when cells are subjected to chronic oxidative stress. SIRTs are shown in the cellular compartment where they are most commonly encountered. SIRT1S and SIRT3L are not targeted to autophagic degradation, but rather act antiapoptotic (SIRT1S) by pinning cytochrome C (CytC), or enhance compensatory mechanism to satisfy bioenergetics demands.

Previous studies have revealed that SIRT1 becomes a target of autophagy in aged tissues, causing a decline in protein level [8]. We were able to confirm this not only for SIRT1 but for all SIRTs with the exception of the minor isoforms SIRT1S and SIRT3L (Fig. 6). The surprising necessity of autophagy to degrade SIRTs may be related to a conserved tetrathiolate. Indeed, high levels of free Zn^2+^ have been observed in autolysosomes, and cells starved for Zn^2+^ demonstrate increased autophagy of Zn^2+^ binding proteins [49, 50]. Since different SIRTs occur more frequently in different cellular compartments, a future approach, which may allow distinguishing between mitophagy and nucleophagy may help gain an even better understanding of these processes. Meanwhile, future projects may also look at different knockout systems (e.g. ATG5, ATG7, or LC3B) to further understand how and when the SIRTs are transferred into the autophagosomes.

Interestingly, not all SIRTs have been shown to exhibit decreased activity after being exposed to oxidation [38], yet we have found all of their major forms to be subjected to autophagic degradation. Thus, their increased degradation may be decoupled from protein activity. Their persisting activity and a lack of carbonylation in most SIRTs would berate a clean damage-related response. Interestingly, this persisting activity was restricted to mostly mitochondrial SIRTs, SIRT3 and SIRT5, despite their Zn^2+^-tetrathiolate being oxidized [38]. The exact function of this thiolate is still not well understood. However, since all previously tested SIRTs were oxidized, while we observed an increased turnover of all SIRTs, this tetrathiolate may mediate the autophagic degradation of the proteins. The previously observed activity pattern would rather fit the conserved H327 (human SIRT1) having obtained sulfurous neighbors. Indeed, it was confirmed that mutation of the C adjacent to H327 resulted in a functional, ROS-resistant SIRT1 [51]. This further strengthens our hypothesis that activity and degradation of SIRT are regulated independently.

SIRT evolution reveals that these sulfurous neighbors must have developed independently, since class IV SIRTs (SIRT6 and SIRT7) evolved an M downstream, while class I SIRTs (SIRT1 and SIRT2) evolved a C upstream. SIRT3 has lost this C again since it became mitochondrial. Thus, we conclude that SIRT7’s activity may also be susceptible toward oxidation, while SIRT4 would not.

The question remains why cells would deplete their active SIRTs under oxidative stress. Induction of apoptosis appears unlikely since the upregulated cytosolic SIRT1S acts antiapoptotically through the scavenging of free cytochrome C [52]. Additionally, SIRT3L has been shown to regulate transcription in favor of cell survival and activate glycolytic enzymes [53–55]. On the other hand, ROS decreasing the bioavailability of SIRTs would leave the cell’s proteome hyper-acetylated, which could be protective in an oxidative environment in two ways. First, the constant damage to biomolecules would require a rapid replacement. Acetylation-mediated stimulation of transcription may facilitate this. Second, the nucleophilic nature of lysine allows it to cross-link with other amino acids or nucleic acids [56, 57]. Acetyl-lysine, however, is chemically more inert and does not form cross-links.

Oxidative stress propels senescence and is a proven stimulus that leads to neurodegenerative events that may develop into disorders of higher-order like PD. While SIRTs are often considered potential treatment targets to prevent or delay senescence, the question needs to be asked whether this would truly prove beneficial for a cell that suffers from chronic oxidative stress. The conserved mechanism of SIRT degradation independent of their activity we describe here would rather suggest that the cell actively removes SIRTs when facing an oxidative dysbalance. Thus, a possible intervention to prolong SIRT availability through, e.g., ectopic expression, may not be as beneficial as the removal of the oxidation source, which would already reestablish a SIRT homeostasis.

## Methods

### Chemicals

Cell culture materials were purchased from Invitrogen and chemicals from Sigma-Aldrich unless stated otherwise. Used primary antibodies included: anti-SIRT1 (9475, CST); anti-SIRT2 (12672, CST); anti-SIRT3 (2627, CST); anti-SIRT4 (NB100-1406, Novus Biologicals); anti-SIRT5 (8782, CST); anti-SIRT6 (12486, CST); anti-SIRT7 (5360, CST); anti-histone H3 (14269, CST); anti-ubiquitin (3936, CST); anti-tubulin (T9026, Sigma-Aldrich); anti-MAP1LC3B (0260–100, NanoTools) for immunocytochemistry, (L7543, Sigma-Aldrich) for immunoblotting; anti-DNP-KLH (A6430, Invitrogen).

### Cell culture

Lund human mesencephalic (LUHMES) cells were grown as published [18]. Four days post differentiation, cells were treated with small molecules in fresh media: MPP^+^ (10 µM, 48 h), PHT (20 nM, 48 h), rotenone (10 nM, 48 h), carbonyl cyanide-p-trifluoromethoxyphenylhydrazone (FCCP) (1 mM, 48 h, Tocris), paraquat (100 µM, 48 h), BafA1 (500 nM, 4 h, Toronto research chemicals), rapamycin (500 nM, 4 h Enzo Lifesciences), MG132 (10 µM, 24 h, Enzo Lifesciences), ATN-224 (10 µM, 6 h, Cayman chemicals). Starvation occurred with EBSS for 4 h.

### Immunoblotting

Immunoblotting was performed according to published protocols [14, 18]. Primary antibodies (1:1000) were detected by horseradish peroxidase-conjugated secondary antibodies (Dianova, 1:10000) through luminescence and quantified using densitometry. PageRulerTM Prestained Protein Ladder (26617, Thermo Fisher Scientific) was used to determine the molecular weight of proteins.

### Immunocytochemistry

Immunocytochemistry and co-localization analysis were performed according to published protocols [14, 18]. Primary antibodies (1:200) were labeled with secondary antibodies (Dianova, 1:400). Hoechst 33258 (Invitrogen) was used for nuclear counterstaining. Cells were photographed using a confocal LSM (TCS SP5, Leica Microsystems).

### SIRT carbonylation assay

Plasmids encoding human SIRT genes were purchased from GenScript Biotech. HEK-T cells were transfected using calcium phosphate precipitation and harvested in H_2_O after 48 h. About 500 µg protein homogenate were incubated with 20 mM H_2_O_2_ for 1 h at room temperature. Reaction was terminated with 1 mM NaCO_3_ and 1x IP buffer (50 mM Tris-HCl, pH 7.4; 150 mM NaCl; 2 mM EDTA; 0.5 mM EGTA; 1% Triton X-100; 10% glycerol; protease inhibitor; 1 mM dithiothreitol). SIRTs were extracted via immunoprecipitation (antibody dilution 1:50) following standard procedure [14]. Carbonylation of proteins was analyzed via immunoblot according to published protocols [58].

### Phylogenetic analysis

Protein sequences were obtained from UniProt and aligned using Clustal Omega [59, 60]. The phylogenetic tree was constructed using iTOL [61]. Binding sites of NAD^+^, SIRT substrates, and tetrathiolate location were extrapolated using the InterPro software [62].

### Statistical analysis

Depending on data structure, Benjamini and Hochberg adjusted one-way or two-way ANOVA were performed. Post hoc test significances with *p* values <0.05 are indicated by asterisks or hash signs. Results are presented as mean ± SD. Linear regression was used to evaluate significant amino acid shifts in protein evolution. If not stated otherwise, the number of replicates (*n*) = 3.

## Supplementary information

Supplementary Figure Legends

Poly-ubiquitinated proteins accumulate after exposure to MG132

BafA1 controls to Figure 3

ATN-224 has no effect on SIRT protein levels

Phylogeny of the SIRT protein family

## Data Availability

Primary data files and calculations are available upon request from either of the corresponding authors (mbaeken@uni-mainz.de, cbehl@uni-mainz.de).
