# Supplementary material for: The selective degradation of sirtuins via macroautophagy in the MPP+ model of Parkinson’s disease is promoted by conserved oxidation sites
Source: Cell Death Discov. 2021 Oct 12;7:286. doi: 10.1038/s41420-021-00683-x (PMC8511006; doi:10.1038/s41420-021-00683-x)
Supplement: Supplementary file 1 — Supplementary Figure Legends [file 41420_2021_683_MOESM1_ESM.docx]

**Supplementary Figure 1: Poly-ubiquitinated proteins accumulate after exposure to MG132**

**a** Representative immunoblots of all poly-ubiquitinated proteins obtained from differentiated LUHMES cells after 48h treatments with MPP^+^ (10 µM) and PHT (20 nM) with and without MG132 (10 µM) supplementation for 24h. H3 was used as loading control. **b** Bar graph showing poly-ubiquitination levels of the blots presented in a. '*' indicates significant differences compared to the control group, while '#' indicates significant differences compared to the MPP^+^-treated group. Symbol number indicates the grade of significance with * = p < 0.05, and ** = p < 0.01. **c** Bar graphs showing the accumulation of all poly-ubiquitinated proteins after MG132 mediated block of proteasomal degradation seen in the blots presented in a.

**Supplementary Figure 2: BafA1 controls to Figure 3**

Representative LSM images of differentiated LUHMES cells treated after 48h treatment with MPP^+^ (10 µM) and/or PHT (20 nM) without BafA1 supplementation with 63x magnification. Stained SIRTs are shown in red and LC3B in green. Scale bars represent 10 µm.

**Supplementary Figure 3: ATN-224 has no effect on SIRT protein levels**

**a** Representative immunoblots of all SIRTs obtained from differentiated LUHMES cells after 6h treatment with ATN-224 (10 µM) with and without BafA1 (500 nM) supplementation for 4h. H3 was used as loading control. **b** Bar graphs showing SIRT protein levels after treatments seen in the blots presented in a.

**Supplementary Figure 4: Phylogeny of the SIRT protein family**

Phylogenetic tree based on protein evolution of SIRT-like NAD^+^ dependent deacylase in eukaryotes and prokaryotes. Colors separate proteins by SIRT-class and phylum.
