# Supplementary figures and images for: The selective degradation of sirtuins via macroautophagy in the MPP+ model of Parkinson’s disease is promoted by conserved oxidation sites
Source: Cell Death Discov. 2021 Oct 12;7:286. doi: 10.1038/s41420-021-00683-x (PMC8511006; doi:10.1038/s41420-021-00683-x)

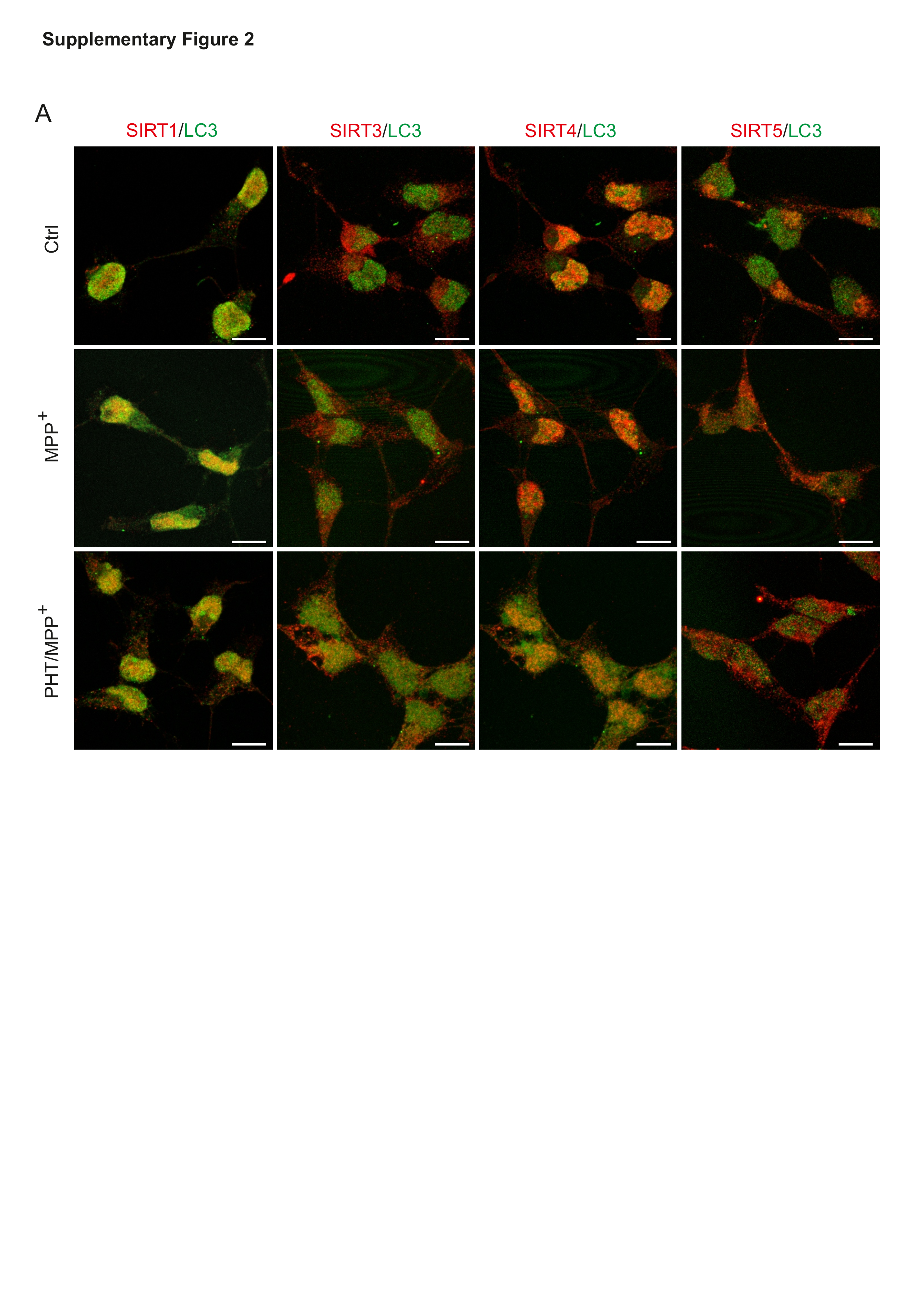

Supplement: Supplementary file 3 — BafA1 controls to Figure 3 [file 41420_2021_683_MOESM3_ESM.tif]

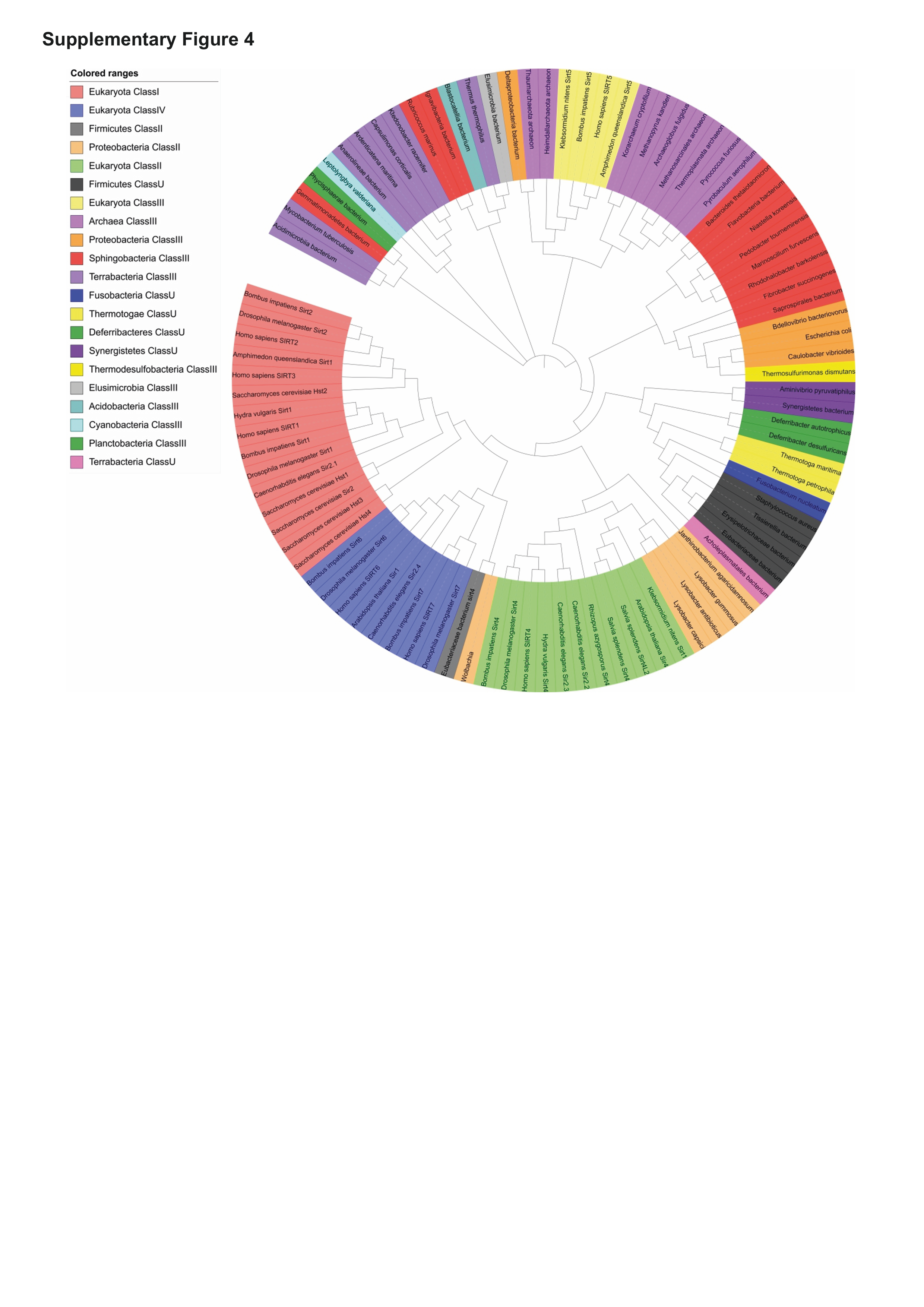

Supplement: Supplementary file 5 — Phylogeny of the SIRT protein family [file 41420_2021_683_MOESM5_ESM.tif]
